# Antiarrhythmic Drugs for Atrial Fibrillation: Selected Features of Ventricular Repolarization That Facilitate Proarrhythmic Torsades de Pointes and Favor Inpatient Initiation

**DOI:** 10.19102/icrm.2021.120704

**Published:** 2021-07-15

**Authors:** James A. Reiffel

**Affiliations:** ^1^Columbia University, New York, NY, USA

**Keywords:** Antiarrhythmic drugs, atrial fibrillation, repolarization, torsades de pointes

## Abstract

The management approaches to patients with atrial fibrillation (AF) include rhythm-control strategies for those patients who are symptomatic despite rate control and for selected others in whom sinus rhythm is necessary for reasons beyond current symptoms (including commercial pilots, those who are felt likely to develop symptoms as comorbidities progress, and more). First-line therapies among the rhythm-control options are antiarrhythmic drugs (AADs). For many AADs, their initiation in-hospital is either a requirement or strongly advised— especially when the patient is in AF. This article explores some of the rationale behind this requirement to give clinicians a better understanding of the reasons for this undesired inconvenience.

## Background

The management strategy for patients with atrial fibrillation (AF) involves four major components: control of the ventricular rate, prevention of thromboembolism, restoration of sinus rhythm with minimization of recurrent AF (rhythm control), and management of the underlying comorbidities that promote AF and/or impair outcome.^1,2^ With respect to rhythm control, one or more antiarrhythmic drugs (AADs) are generally employed prior to attempts at ablative therapies and are also commonly employed when ablation has failed. Additionally, when AADs are used for AF, whether prior to or following ablation, the major guidelines^1,2^ stress a safety-first approach. That is, when the ventricular response is controlled and appropriate oral anticoagulation has been instituted and stabilized, AF patients should have a markedly reduced risk of mortality or major morbidity from the AF itself. Therefore, attempts at rhythm control should use AADs with a low risk profile prior to the pursuit of approaches that may be more effective but carry a greater risk, such as amiodarone or ablation, as well as prior to a repeat ablation procedure. To directly quote the 2016 European Society of Cardiology (ESC) guidelines, as an example,^2^ “amiodarone is more effective in preventing AF recurrences than other AADs, but extracardiac toxic effects are common and increase with time. For this reason, other AAD should be considered first.” Because of the proarrhythmic risk, major guidelines^1,2^ and/or package inserts indicate that, for some of these AADs, initiation should occur in the hospital under electrocardiogram (ECG) monitoring. The purpose of this article is to highlight for clinicians the major reasons behind such recommendations so as to minimize the risk of proarrhythmic patient death that could result from unmonitored outpatient initiation.

No AAD is entirely free of notable risks. Most prominently, these include both organ toxicity and proarrhythmic ventricular tachyarrhythmias that may cause syncope or death. Organ toxicity is a serious concern with amiodarone but appears to be much less of an issue with other AADs (though it also was with quinidine and procainamide).^3–6^ Ventricular proarrhythmia,^3,5,7–14^ in the form of monomorphic ventricular tachycardia (VT) [which may degenerate into ventricular fibrillation (VF)],^15,16^ is mostly a concern with inhibitors of the fast sodium current, eg, class I AADs. Reentry is the mechanism for class I AAD-induced monomorphic VT, which almost always occurs in the setting of underlying anatomic or histopathologic left ventricular (LV) abnormalities or ischemia that modify conduction patterns within the ventricles so as to facilitate reentrant loops. The risk of developing monomorphic VT as a proarrhythmic response to a class I AAD in the setting of a ventricular structural or functional disorder appears to be greatest with class IC AADs, which have a more potent effect on sodium channel blockade than do class IA or class IB AADs. Ventricular proarrhythmia, in the form of torsades de pointes (TdP) polymorphic VT (which also may degenerate into VF), is primarily a concern with drugs that prolong ventricular repolarization—specifically, class IA AADs and class III AADs. Herein, sotalol—and, even more so, dofetilide—carries a much greater risk than the mixed channel agents often classified predominantly as class III, which are amiodarone and dronedarone. Amiodarone and dronedarone are more complex agents that have properties other than those of class II and class III drugs, which might lessen the risk of TdP. Moreover, they reduce the dispersion of repolarization in the ventricular myocardium, which lessens the likelihood of TdP development, in contrast with the increase in dispersion that occurs with sotalol and dofetilide. Because of the TdP risk, in Europe, dofetilide has not yet been approved for use and, in the 2020 ESC guidelines, sotalol has been reduced from a class I level of recommendation to a class IIb.^17^ (“Sotalol may be considered for long-term rhythm control in patients with normal LV function or with ischaemic heart disease if close monitoring of the QT interval, serum potassium level, CrCl, and other proarrhythmia risk factors is provided.”)

The risk of TdP with a class IA or class III AAD is not entirely an event that occurs by chance. Rather, there are factors that are known to enhance the risk. Among the most prominent are those that are modifiable (hypokalemia, hypomagnesemia, or co-administration of drugs that prolong repolarization), those that may be modifiable (bradycardia, ventricular hypertrophy, altered drug clearance), and those that are inherited (female sex and specific gene defects)^7–11^ **(Table 1)**. The common features of these are enhancement of the potential for early afterdepolarizations (EADs) and reentry during ventricular repolarization. Among the electrophysiologic factors that contribute to TdP, prolongation of ventricular repolarization is a major contributory component, especially if there is also increased dispersion of repolarization (represented on the ECG as the difference between the longest and the shortest QT intervals).^7–11^

The issue of the relationship between prolonged ventricular repolarization and an increased risk for TdP with potassium channel–blocking AADs has been known for years, but some components of this relationship are particularly relevant. It is these features that the rest of this article will address, as their recognition may help to mitigate the risk and as they are important with respect to the need for hospitalization when class IA and III AADs are initiated in a patient who is in AF.

## Ventricular repolarization

### Bradycardia and action-potential duration

Repolarization duration in the His–Purkinje tissue and in ventricular myocytes reflects the length of their action potential and their action potential length is, in a significant part, dependent upon the previous R–R cycle length (ie, both the immediately preceding R–R interval and the few before it).^18,19^ The longer the preceding R–R interval, the longer the next myocyte action-potential duration will be. In the presence of a reduced outward potassium current during the action-potential plateau and phase 3, whether due to a genetic defect, changes associated with ventricular hypertrophy, or drug suppression of potassium channel conductance (particularly Ik_r_), the longer the myocyte action potential and the greater the likelihood of development of EADs, which can trigger TdP.^7–11^

Upon recognizing this relationship, it should be clear that bradycardia will be associated with an increase in action-potential duration. Accordingly, the pause that occurs with the termination of AF, whether spontaneously or via cardioversion (direct-current or drug-induced), is most likely the longest R–R cycle the patient will encounter. This should reasonably be associated with the moment of greatest risk for TdP in a patient taking a class IA or III AAD. Given this relationship and risk, it should be no surprise that the initiation of such drugs in a patient in AF, which might terminate following drug initiation, is generally advised to occur in-hospital under monitored conditions.^1,2,20^ Notably, via indirect comparisons, as the risk of TdP appears to be lower with sotalol than with dofetilide, AF guidelines have not broadly mandated in-hospital initiation of sotalol if the patient is in sinus rhythm and has no other risk markers for TdP, such as hypokalemia, hypomagnesemia, diuretic therapy, bradycardia/tachy–brady syndrome, and renal impairment. For example, the 2006 American College of Cardiology (ACC)/American Heart Association (AHA)/ESC guidelines^20^ state that, “as long as the baseline uncorrected QT interval is less than 460 ms, serum electrolytes are normal, and risk factors associated with class III drug–related proarrhythmia are considered, sotalol may be initiated in outpatients with little or no heart disease. It is safest to start sotalol when the patient is in sinus rhythm.” In contrast, those same 2006 guidelines also state that “quinidine, procainamide, and disopyramide should not be started out of [the] hospital and out-of-hospital initiation of dofetilide is not currently permitted.” Further in contrast [and surprisingly and unfortunately, in my opinion], while the 2014 AHA/ACC/Heart Rhythm Society (HRS) guidelines^1^ specifically state that “dofetilide therapy should not be initiated out of hospital owing to the risk of excessive QT prolongation that can cause torsades de pointes,” they no longer mention where sotalol should be started—moreover, neither do the 2019 AHA/ACC/HRS guideline updates^21^ or the 2020 ESC guidelines.^17^ Interestingly, and in contrast, the package inserts for sotalol, dofetilide, and quinidine each suggest in-hospital initiation at all times and include the specific dose ranges not to be exceeded and the appropriate QT and renal status criteria to guide dosing.^22,23^ In association with the concern regarding TdP and its relationship to bradycardia, one should always consider any previously demonstrated effect(s) on the sinus node of the drug(s) being used to control the ventricular response during AF and should adjust them accordingly as needed if sinus rhythm is to be restored with a QT interval–prolonging AAD. Because of its intrinsic sympathomimetic activity, pindolol is the least likely of all the available β-blockers to suppress sinus rates even though it can be effective in depressing atrioventricular (AV) nodal conduction during AF^24^ and, as such, may be the safest to use when a β-blocker is added to dofetilide if sinus rate slowdown is a concern.

### Repolarization reserve

Why is it that not all patients given a class IA or III AAD develop significant action-potential lengthening and, with it, a prolonged QT or corrected QT (QTc) interval? This can, at least in part, be explained by the concept of repolarization reserve.^25–27^ We each have multiple ion channels that contribute to ventricular repolarization, including several different potassium currents. If the administered AAD, such as dofetilide, impairs one type of current, such as Ik_r_, but the others remain capable of increasing their conduction of potassium outwardly, the net effect will be no significant increase in ventricular action-potential duration and, therefore, QT interval. The ability for nonblocked channels to increase their potassium-carrying current reflects their “reserve,” and hence the term repolarization reserve.^25–27^ If, however, the other channels cannot adequately increase their reserve, usually because of the individual’s gene-determined pattern^28,29^ but possibly due to the effect of multiple drugs taken in combination, then action-potential lengthening will occur, and, with it, an increased risk for TdP. This, too, should become apparent relatively soon after a class IA or III AAD is started and, hence, supports the need for the advised in-hospital period of observation. Repolarization reserve is also linked to the observation that many patients who develop TdP when given QT interval–prolonging drugs do not show any QT prolongation on their ECGs in the absence of such medications.

## The effects of adaptation

Adaptation refers to the observation that a sustained change in the ventricular rate will be associated with a change in the duration of repolarization,^30,31^ though it may occur slowly.^31–33^ For example, following cardioversion of a patient who has been in AF with a ventricular rate significantly faster than the postconversion sinus rhythm rate, the action-potential duration, as reflected by the QT or QTc interval, will be longer immediately after cardioversion as compared with later on, despite no change in the sinus rate. This is due to the initial “relative bradycardia” of sinus rhythm in contrast with the ventricular rate during AF. In this circumstance, the need for an absolutely bradycardic post-AF rate is not necessary. Of note, however, this may only be apparent in circumstances where the QT interval is initially prolonged, as with a widened QRS or in the presence of a QT-prolonging medication. Our laboratory demonstrated the effect of abrupt rate slowing on ventricular repolarization and its evolution some years ago when patients with drug-refractory rapid ventricular rates in AF were treated with AV nodal ablation and implantation of a permanent ventricular pacemaker.^32^ The QTc interval during pacing was longest shortly after cardioversion than at later times. More specifically, 12-lead ECGs during right ventricular pacing at rates of 60, 80, 100, and 120 bpm were recorded at 30 minutes, 24 hours, one week, and one month after AV node ablation because of refractory AF in 15 patients. The mean ventricular rates during AF in these patients preablation were 93 bpm at rest and 156 bpm during activity. In each case, the QT interval at 24 hours, one week, and one month was shorter than that at 30 minutes at each pacing rate, and there were no statistically significant differences between the QT intervals at 24 hours and later time points. In five control patients without AF who received pacemakers for bradycardic indications, there were no statistically significant changes in the QT interval at any time point at identical pacing rates. Similar observations were subsequently made by Grom et al.^33^ Also, related findings were reported by Lenhoff et al.^34^ in a comparison of sotalol versus metoprolol; these authors studied 208 patients (104 on sotalol and 104 on metoprolol, using maximally tolerable doses) who maintained sinus rhythm during the first week after cardioversion for AF. Twelve-lead ECGs were recorded at one hour and one week postcardioversion. One hour postcardioversion, the QTc interval was significantly longer in the sotalol group than in the metoprolol group (465 vs. 423 ms). After one week, the QTc had been shortened by 23 ms in the sotalol-treated patients but remained unchanged in the metoprolol-treated patients.

Here, too, as the QT will be longer after cardioversion than it will be at the same rate at later points in time, observation of the patient early after cardioversion for the TdP risk is advised.

## Sex

When considering TdP and sex simultaneously, female sex is the one that almost always comes to mind. With virtually every drug studied that has been associated with TdP, its incidence has been revealed as being approximately twice as high in women than in men once puberty has passed.^35–37^ Moreover, the effect of postpubertal gender on ventricular repolarization does not change with menopause.^38^ While there appear to be several mechanisms at play with respect to this relationship, their end-effect is that women have longer QT and QTc intervals than men. Thus, when starting an AAD for AF, it would seem logical that women should be monitored more closely than men (such as by more frequent analysis of ECG intervals, renal function, and electrolytes and the adoption of longer time periods between dose escalation and shorter times to posthospital follow-up). However, this is not specifically stated in any AF guidelines.^1,2,20,21^

Interestingly, the relationship may not simply be because the longest QT interval on 12-lead ECGs from women is longer than that from men,^39–41^ but, also of note, our group reported that the shortest QT interval on 12-lead ECGs from women is likewise longer than the shortest QT interval from men.^41^ Moreover, the difference between the longest and shortest QT intervals in men is greater than in women. In other words, the shortest QT interval in women is significantly longer than the shortest QT interval in men and the shortest QT interval in women minus that in men is greater than the difference between the longest QT interval in women minus that in men. If TdP is at least in part a probability function related to the length of action potentials within the myocardium and a functional threshold for the shortest action potential at which EADs may begin to develop, women have more action potentials near that threshold in the baseline state than men do, and it might take less drug to cause EADs to develop in women than in men. Paradoxically, this is one circumstance where QT dispersion is less in the group at greater risk.

The “flip side” of such observations is that the AF guidelines do not specifically state a need to monitor women more closely than men when starting an AAD or that there are more men with AF than women at any age. Concordantly, while the incidence of TdP development in women is greater than that in men, the actual number of men who develop TdP on an AAD is not less than that of women. For example, we examined proarrhythmic risks a few decades ago in the Electrophysiologic Study Versus Electrocardiographic Monitoring (ESVEM) trial,^33^ which explored the effect of seven AADs in the management of sustained VT/VF.^42,43^ As one of the ESVEM investigators and writing committee members, during our assessment of adverse risks and events, I used the ESVEM database to assess the frequency of excess QT prolongation (> 500 ms) and TdP events by gender that occurred when any or sequential AADs were given to the ventricular tachyarrhythmia patients enrolled. These drugs included imipramine, mexiletine, pirmenol, procainamide, propafenone, quinidine, and sotalol—that is, several class IA and III AADs. Of the 486 patients who received one or more AADs, 88% were men, the mean age was 65 years, 85% had coronary artery disease, and the mean LV ejection fraction was 32%. Excess QT prolongation occurred in nine of 426 men (2.1%) versus three of 60 women (5%), and TdP occurred in eight of 426 men (1.9%) versus two of 60 women (3.3%). Though these numbers were too small to reach statistical significance and we elected not to publish them at that time, clearly, the trends favored the historical impression of a higher numerical risk in women than in men. However, 75% of the excess QT prolongation and 80% of the TdP observed in the study occurred in men.

## Concluding thoughts

AF is increasingly occupying the focus of clinical cardiologists as an entity requiring attention. In many patients with AF, AAD therapy is required. Unfortunately, with some of our AADs and in some of our patients, in-hospital initiation of the AAD is required. Most patients would prefer to avoid the inconveniences and costs associated with hospitalizations. Moreover, judging by the overuse of amiodarone as determined by prescription monitoring—a drug that is not approved by the United States Food and Drug Administration for AF—most physicians also prefer not to have to hospitalize their patients just to begin an AAD. Nonetheless, our current AF guidelines for rhythm-control approaches in AF patients are based upon a safety-first strategy, and this strategy requires in-hospital initiation for some drugs in some patients **(Table 2)**. The material in this article is meant to provide readers with some additional insight behind the in-hospital requirements.

## Figures and Tables

**Table 1:** Factors That Clinicians Should Remain Aware of with Respect to AAD-induced Proarrhythmia

Drug Class	Primary Proarrhythmia Type	Contributing Factors
IA and IC	Reentrant ventricular tachycardia	Comorbidities that result in regions of slow conduction, unequal conduction, and unidirectional block of conduction in ventricular myocardium, including scar, ischemia, and infiltration
IA and III	TdP	Factors that lengthen the QT interval besides the AAD, including bradycardia (absolute, relative, and/or abrupt), concomitant QT-prolonging drugs, impaired repolarization reserve, ventricular hypertrophy, impaired drug clearance, female sex, and specific gene defects
IA and III	TdP	Factors that facilitate the development of afterdepolarizations; in addition to the factors noted above, which can result in a prolonged QT interval, hypokalemia and hypomagnesemia can facilitate the development of TdP

AAD: antiarrhythmic drug; TdP: torsades de pointes.

**Table 2:** Considerations Concerning AADs with Respect to In-hospital Initiation^1,2,17,20,21^

**For All Drugs That Can Increase the QT Interval:**	
•	Correct electrolyte abnormalities, especially hypokalemia and hypomagnesemia
•	Correct bradycardia, if possible (consider pacemaker if necessary)
•	Discontinue or reduce dosage of concomitant drugs (if possible) that can impair metabolism and/or clearance of the AAD and/or that can themselves increase the QT interval
•	Avoid using the AAD if the QT or QTc interval is prolonged at baseline or if the patient has a known genotype associated with QT-interval prolongation
•	Use extra caution (eg, consider starting with a lower dose or incrementing doses more slowly) in women
•	Discontinue the AAD if undue QT-interval lengthening occurs (which can be due to sex or heart rate and drug-specific—check the package inserts) and monitor the ECG intervals at least until a pharmacologic steady state is achieved
**For Specific Drugs:**	
•	Dofetilide (or quinidine): in-hospital initiation for all patients, as well as for increments in dosage.
•	Sotalol: in-hospital initiation for all patients if in atrial fibrillation; can consider outpatient initiation if nonbradycardic; male; and/or with normal electrolytes, normal baseline QT interval, normal renal function, and no concomitant interacting medications

AAD: antiarrhythmic drug; QTc: corrected QT.

## References

[1] January CT, Wann S, Alpert JS (2014). 2014 AHA/ACC/HRS guideline for the management of patients with atrial fibrillation: executive summary: a report of the American College of Cardiology/American Heart Association task force on practice guidelines, and the Heart Rhythm Society. Circulation.

[2] Kirchoff P, Benussi S, Kotecha D (2016). 2016 ESC guidelines for the management of atrial fibrillation developed in collaboration with EACTS. Eur Heart J.

[3] Reiffel JA (2000). Drug choices in the treatment of atrial fibrillation. Am J Cardiol.

[4] Podrid PJ (1991). Safety and toxicity of antiarrhythmic drug therapy: benefit versus risk. J Cardiovasc Pharmacol.

[5] Camm AJ (2006). Safety considerations in the pharmacological management of atrial fibrillation. Int J Cardiol.

[6] Patton KK, Page RL (2007). Pharmacological therapy of atrial fibrillation. Expert Opin Investig Drugs.

[7] Trinkley KE, Page RL, Lien H, Yamanouye K, Tisdale JE (2013). QT interval prolongation and the risk of torsades de pointes: essentials for clinicians. Curr Med Res Opinion.

[8] Schwartz PJ, Woosley RL (2016). Predicting the unpredictable: drug-induced QT prolongation and torsades de pointes. J Am Coll Cardiol.

[9] Naccarelli GV, Wolbrette DL, Luck JC (2001). Proarrhythmia. Med Clin North Am.

[10] Roden DM, Anderson ME (2006). Proarrhythmia. Handb Exp Pharmacol.

[11] Frommeyer G, Eckardt L (2016). Drug-induced proarrhythmia: risk factors and electrophysiological mechanisms. Nat Rev Cardiol.

[12] Turner JR, Karnad DR, Cabell CH, Kothari S (2017). Recent developments in the science of proarrhythmic cardiac safety of new drugs. Eur Heart J Cardiovasc Pharmacother.

[13] El-Sherif N, Turitto G, Boutjdir M (2019). Acquired long QT syndrome and electrophysiology of torsades de pointes. Arr Electrophysiol Rev.

[14] Murakawa Y (2011). Focal and reentrant mechanisms of torsades de pointes: EAD, reentry, or chimera?. J Arrhythmia.

[15] Xie Y, Grandi E, Bers DM, Sato D (2014). How does beta-adrenergic signalling affect the transitions from ventricular tachycardia to ventricular fibrillation. Europace.

[16] Ashihara T, Yao T, Namba T (2002). Afterdepolarizations promote the transition from ventricular tachycardia to fibrillation in a three-dimensional model of cardiac tissue. Circ J.

[17] Hindricks G, Potpata T, Dagres N (2020). 2020 ESC guidelines for the diagnosis and management of atrial fibrillation developed in collaboration with the European Association of Cardio-Thoracic Surgery (EACTS) [published online ahead of print August 29, 2020]. Eur Heart J.

[18] Anderson ME (2006). QT interval prolongation and arrhythmia: an unbreakable connection?. J Intern Med.

[19] Vaughn Williams EM (1982). QT and action potential duration. Br Heart J.

[20] Furster V, Ryden LE, Cannom DS (2006). ACC/AHA/ESC 2006 guidelines for the management of patients with atrial fibrillation—executive summary. Circulation.

[21] January CT, Wann LS, Calkins H (2019). 2019 AHA/ACC/HRS focused update of the 2014 AHA/ACC/HRS guideline for the management of patients with atrial fibrillation: a report of the American College of Cardiology/American Heart Association task force on clinical practice guidelines and the heart Rhythm Society in collaboration with the Society of Thoracic Surgeons. Circulation.

[22] United States Food and Drug Administration BETAPACE AF^®^ (sotalol HCl).

[23] United States Food and Drug Administration TIKOSYN^®^ (dofetilide) capsules.

[24] Reiffel JA (1992). Improved rate control in atrial fibrillation. Am Heart J.

[25] Roden DM (2005). Long QT syndrome: reduced repolarization reserve and the genetic link. J Intern Med.

[26] Varro A, Baczko I (2011). Cardiac ventricular repolarization reserve: a principle for understanding cardiac drug-related proarrhythmic risk. Br J Pharmacol.

[27] Roden DM (2008). Repolarization reserve: a moving target. Circulation.

[28] Mazzanti A, Maragna R, Vacanti G (2018). Interplay between genetic substrate, QTc duration, and arrhythmia risk in patients with long QT syndrome. J Am Coll Cardiol.

[29] Shimizu W (2010). How the knowledge of genetic “makeup” and cellular data can affect the analysis of repolarization in surface electrocardiogram. J Electrocardiol.

[30] Bergfeldt L, Lundahl G, Bergqvist G, Vahedi F, Gransberg L (2017). Ventricular repolarization and dispersion adaptation after atropine induced rapid heart rate increase in healthy adults. J Electrocardiol.

[31] Bueno-Orovio A, Hanson BM, Gill JS, Taggart P, Rodriguez B (2014). Slow adaptation of ventricular repolarization as a cause of arrhythmia?. Methods Inf Med.

[32] Dizon J, Blitzer M, Rubin D (2000). Time dependent changes in duration of ventricular repolarization after AV node ablation: insights into the possible mechanism of postprocedural sudden death. Pacing and Clin Electrophys.

[33] Grom A, Faber TS, Brunner M, Bode C, Zehender M (2005). Delayed adaptation of ventricular repolarization after sudden changes in heart rate due to conversion of atrial fibrillation: a potential risk factor for proarrhythmia?. Europace.

[34] Lenhoff H, Darpo B, Ferber G, Rosenqvist M, Frick M (2016). Reduction over time of QTc prolongation in patients with sotalol after cardioversion of atrial fibrillation. Heart Rhythm.

[35] Makkar RR, Fromm BS, Steinman RT, Meissner MD, Lehmann MH (1993). Female gender as a risk factor for torsades de pointes associated with cardiovascular drugs. JAMA.

[36] Chorin E, Hochstade A, Viskin S (2016). Female gender as an independent risk factor of torsades de pointes during acquired atrioventricular block. Heart Rhythm.

[37] Bednar MM, Harrigan EP, Ruskin JN (2002). Torsades de pointes associated with nonantiarrhythmic drugs and observations on gender and QTc. Am J Cardiol.

[38] Dogan U, Dougan NU, Basarir AO (2012). P-wave parameters and cardiac repolarization indices: does menopausal status matter. J Cardiol.

[39] Salama G, Bett GC (2014). Sex differences in the mechanisms underlying long QT syndrome. Am J Physiol.

[40] Al-Khatib SM, LaPointe NMA, Kramer JM, Califf RM (2003). What clinicians should know about the QT interval. JAMA.

[41] Kassotis J, Costeas C, Bedi AK, Tolat A, Reiffel J (2000). The effects of aging and gender on QT dispersion in an overtly healthy population. Pacing Clin Electrophysiol.

[42] Mason JW, Marcus FI, Bigger JT (1996). A summary and assessment of the findings and conclusions of the ESVEM trial. Prog Cardiovasc Dis.

[43] The ESVEM Investigators (1989). The ESVEM trial: electrophysiologic study versus electrocardiographic monitoring for selection of antiarrhythmic therapy of ventricular tachyarrhythmias. Circulation.

